# Treatment of refractory generalized granuloma annulare with oral vitamin E and topical tea tree oil

**DOI:** 10.1016/j.jdcr.2022.06.004

**Published:** 2022-06-20

**Authors:** Rebecca Colwell, Daniel D. Bennett, Justin Endo

**Affiliations:** University of Wisconsin School of Medicine and Public Health

**Keywords:** granuloma annulare, Melaleuca, tea tree oil, tocopherol, vitamin E, GA, granuloma annulare, IL, interleukin, TTO, tea tree oil

## Introduction

Generalized granuloma annulare (GA) is an uncommon form of GA that is recalcitrant to treatment.^1^In contrast to localized GA, the generalized form is characterized by widespread annular plaques or papules over the trunk and extremities.^1^ Although the lesions are frequently asymptomatic, the lesions can be disfiguring and decrease the patient’s quality of life. Therefore, extensive disease might justify more aggressive treatment. We report a patient with generalized GA resistant to conventional treatments who experienced dramatic improvement with oral vitamin E and topical tea tree oil (TTO).

## Case

**Fig 1:**
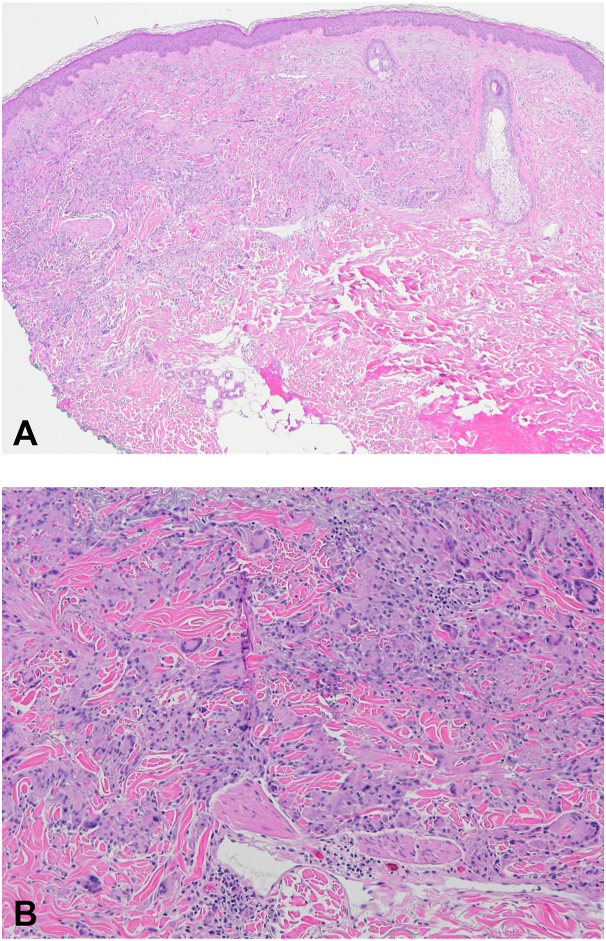
“Granuloma annulare.” Superficial-to-mid dermal palisaded granulomatous infiltrate with multinucleated giant cells. **A,** Hematoxylin and eosin stain, original magnification ×40. **B,** Hematoxylin and eosin stain, original magnification ×100.

**Fig 2:**
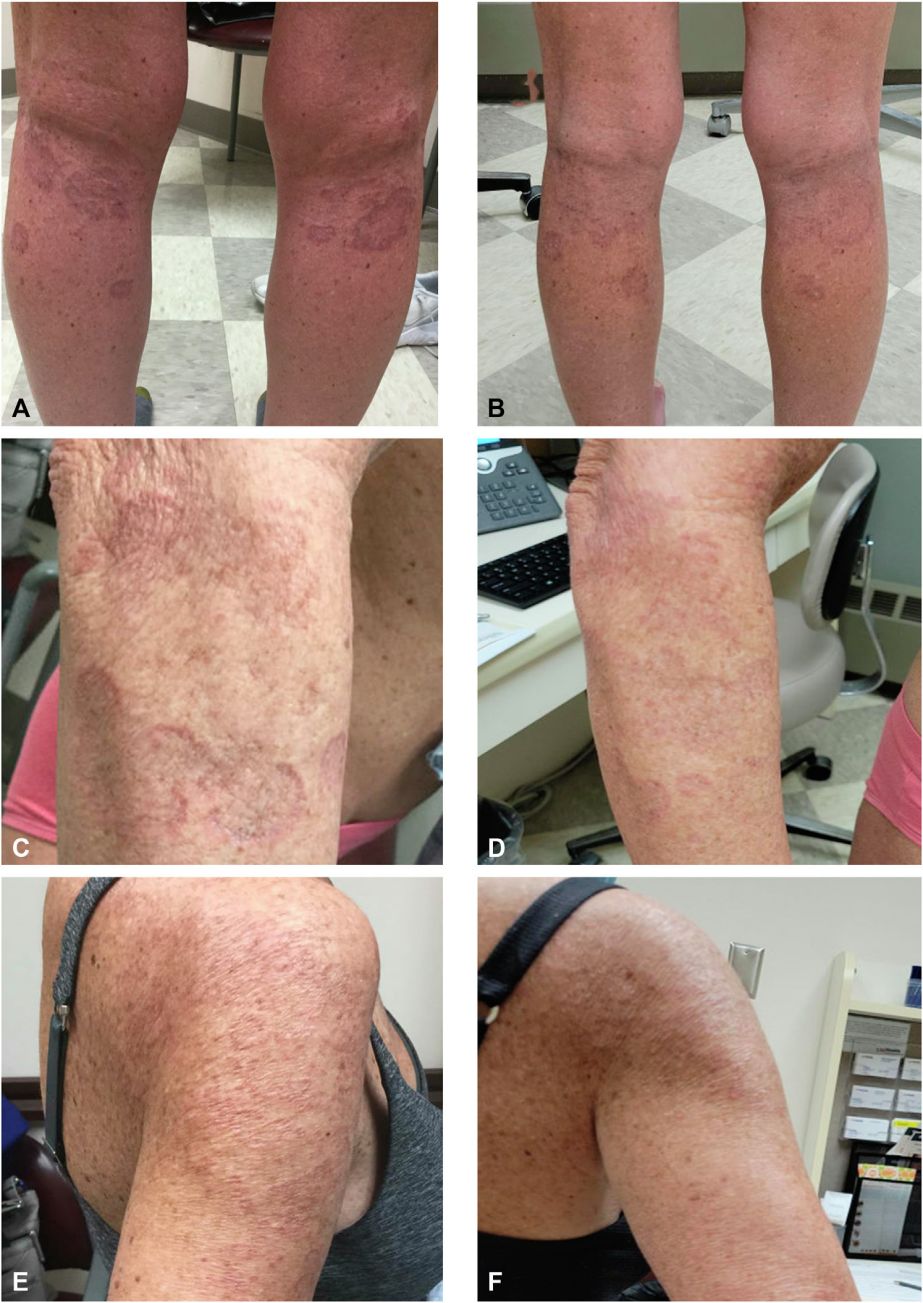
“Granuloma annulare.” Annular plaques prior to initiation of vitamin E and tea tree oil (**A**, **C**, **E**). Annular plaques after 3 weeks of treatment with oral vitamin E and topical tea tree oil (**B**, **D**, **F**).

A 48-year-old woman presented with a mildly pruritic rash over the forearms and elbows. The biopsy showed palisaded granulomatous dermatitis. She was treated with betamethasone dipropionate 0.05% lotion for about 1 year and pimecrolimus 1% cream without improvement. She presented 2 years later with spreading annular plaques over the right leg, left shoulder, and bilateral forearms. Two additional biopsies were performed. All 3 of this patient's biopsies demonstrated very similar features with a superficial-to-mid dermal palisaded granulomatous infiltrate (Fig 1, *A* and *B*). Multinucleated giant cells were conspicuous in all 3. One specimen demonstrated focal mucin deposition using a colloidal iron stain, while 2 failed to show significant mucin with Alcian blue. One biopsy was also associated with focally conspicuous eosinophils, indicating possible drug-induced GA. She stopped furosemide and spironolactone and tried calcipotriene 0.005% cream without improvement. She had modest improvement with narrowband ultraviolet B, but this was not considered a safe long-term option because of long-standing actinic damage. One year later, she presented with additional reddish-brown annular papules and plaques on the back, bilateral upper extremities, and bilateral lower extremities (Fig 2, *A*, *C*, *E*). She was treated with hydroxychloroquine and minocycline for almost 1 year without improvement. She discontinued these on her own and started oral vitamin E 180 mg once daily and topical TTO twice daily for 1 week and then transitioned to once daily. Within 3 weeks, most of her lesions flattened and faded (Fig 2, *B*, *D*, *F*). Notably, the lesions only improved in areas treated with topical TTO. She was recommended to stop the vitamin E due to potential long-term risks and continue the topical TTO.

## Discussion

Generalized GA is challenging to manage. Patients who have not responded to traditional skin-directed or systemic treatments may explore unconventional therapies. Some of these less commonly utilized treatments for GA include vitamin E and pentoxifylline.^2^ Vitamin E, or tocopherol, has been used for treating GA since the 1940s.^3^ Many patients view alternative treatments favorably, and this is particularly relevant in patients who have failed to respond to conventional therapies.^4^

Vitamin E provides anti-inflammatory and immune-regulation effects that can be beneficial for multiple cutaneous conditions.^5^ It sequesters free radicals and decreases inflammatory mediators such as tumor necrosis factor, metalloproteinases, and malondialdehyde.^6^ It is often well tolerated and can be beneficial in patients who prefer a “natural” treatment. When considering initiation of this treatment, it is important to consider the stability of the form used. Topical formulations oxidize quickly when exposed to light and air. Additional considerations include interactions with vitamin K–dependent clotting factors in patients taking large doses of 1000 IU of oral vitamin E.^2^^,^^7^

Treatment with α-, β-, γ-, and δ-tocopherols was found to be beneficial in patients with GA.^3^ Similarly, a compound containing only α-tocopherol was used and found to be beneficial in 10 out of 13 patients with GA, including a patient with 6 years of symptoms.^3^ The therapeutic effect of vitamin E on GA is likely due to its anti-inflammatory effects.

The second therapy of interest, TTO (*Melaleuca alternifolia*), has multiple dermatologic applications, including wound healing, regulating wheal and flare, and the treatment of seborrheic dermatitis, chronic gingivitis, acne, and scabies.^8^ TTO is an essential oil distilled from parts of *Melaleuca alternifolia*, *Melaleuca dissitiflora*, and *Melaleuca linariifolia* plants.^9^ It has anti-inflammatory, antibacterial, antioxidant, antiviral, antifungal, antiprotozoal, and antitumor properties.^8^ Terpinen-4-ol, one of the bioactive components of TTO, contributes antimicrobial and anti-inflammatory properties.^8^ The mechanism of action of terpinen-4-ol occurs through reducing tumor necrosis factor, interleukin (IL)-1, IL-8, IL-10, and prostaglandin E2. It modulates vasodilation, plasma extravasation, and superoxide production in monocytes.^8^

TTO is available over the counter as an active ingredient in many products, touted for its anti-inflammatory and antimicrobial effects. TTO has not previously been reported to be beneficial in the treatment of GA.^4^^,^^8^ The therapeutic effect of TTO on GA is likely due to its anti-inflammatory effects.

Adverse effects of TTO include toxicity when ingested at high doses, skin irritation, allergic contact dermatitis, systemic contact dermatitis, linear immunoglobulin A disease, erythema multiforme–like reactions, and systemic hypersensitivity reactions.^8^ Allergic contact dermatitis to TTO occurs in response to oxidation products resulting from improperly stored oil.^4^

Generalized GA is often recalcitrant to therapy. Spontaneous remission can occur and may have influenced this patient’s case. However, this patient had spreading disease over years. Data regarding the efficacy and safety of alternative medicines can be sparse, leading to slow adaptation of alternative medicines. Patients with generalized GA unresponsive to conventional treatments may benefit from vitamin E and TTO therapies.

## Conflicts of interest

Dr Bennett holds the office of secretary treasurer of the American Academy of Dermatology. The other authors declare no conflict of interest.
